# Eustachian Tube Obstruction Grade as an Independent Determinant of Audiological and Quality-of-Life Outcomes in Pediatric Chronic Adenoiditis: A Retrospective Cohort Study

**DOI:** 10.3390/medicina62071297

**Published:** 2026-07-05

**Authors:** Diana Szekely, Flavia Zara, Raul Patrascu, Cristina Stefania Dumitru, Alina Cristina Barb, Dorin Novacescu, Antonia Armega Anghelescu, Alexia Manole, Dan Iovanescu, Gheorghe Iovanescu

**Affiliations:** 1 Doctoral School, “Victor Babes” University of Medicine and Pharmacy Timisoara, E. Murgu Square, No. 2, 300041 Timisoara, Romania; diana.szekely@umft.ro; 2 Department II of Microscopic Morphology, Discipline of Histology, “Victor Babes” University of Medicine and Pharmacy Timisoara, E. Murgu Square, No. 2, 300041 Timisoara, Romania; flavia.zara@umft.ro (F.Z.); toma.alina@umft.ro (A.C.B.); novacescu.dorin@umft.ro (D.N.); 3 Department of Functional Sciences, “Victor Babes” University of Medicine and Pharmacy Timisoara, E. Murgu Square, No. 2, 300041 Timisoara, Romania; patrascu.raul@umft.ro; 4 Faculty of Medicine and Pharmacy, University of Oradea, 410087 Oradea, Romania; manole.alexia@student.uoradea.ro; 5 Otorhinolaryngology Department, “Victor Babes” University of Medicine and Pharmacy Timisoara, E. Murgu Square, No. 2, 300041 Timisoara, Romania; dan.iovanescu@umft.ro (D.I.); giovanescu@umft.ro (G.I.)

**Keywords:** Eustachian tube dysfunction, chronic adenoiditis, otitis media with effusion, pure tone audiometry, quality of life, pediatric otorhinolaryngology, endoscopic grading, middle ear pressure, mucosal remodeling

## Abstract

*Background and Objectives*: Eustachian tube (ET) dysfunction links adenoidal disease to persistent middle ear dysfunction in children, yet the independent contribution of ET orifice obstruction grade to audiological outcomes and health-related quality of life remains unquantified after adjustment for anatomical and inflammatory confounders. Because conventional anatomical grading (e.g., the Cassano classification) does not directly characterize the degree of ET orifice compromise, it may underestimate the functional threat to middle ear ventilation; this study is the first to quantify the independent predictive value of endoscopic ET obstruction grade. This study aimed to evaluate ET obstruction grade as an independent determinant of hearing thresholds, middle ear pressure, and quality-of-life impairment in children with chronic adenoiditis and otitis media with effusion. *Materials and Methods*: A retrospective cohort of 236 children (aged 3–12 years) was analyzed. ET orifice obstruction was graded endoscopically as none, partial, or complete. Primary outcomes included pure tone average (PTA), middle ear pressure (MEP), and OSA-18 total score. Multivariate linear and logistic regression models were fitted, adjusting for age, sex, Cassano grade, neutrophil-to-lymphocyte ratio (NLR), allergic status, and acute otitis media frequency. The modifying role of mucosal appearance (edematous versus fibrotic/remodeling) on quality-of-life outcomes was also assessed. *Results*: ET obstruction was absent in 42 (17.8%), partial in 114 (48.3%), and complete in 80 (33.9%) children. PTA increased progressively across groups (22.2 ± 5.5 to 36.2 ± 6.7 dB; *p* < 0.001), as did OSA-18 scores (44.9 ± 7.9 to 80.4 ± 10.3; *p* < 0.001). In adjusted analysis, each obstruction increment independently predicted a 5.57 dB PTA increase (95% CI 4.37–6.77; *p* < 0.001), a 14.89-point OSA-18 increase (95% CI 12.87–16.92; *p* < 0.001), and 5.12-fold higher odds of PTA > 30 dB (95% CI 2.84–9.24; *p* < 0.001). Persistent middle ear dysfunction at six months occurred in 7.1%, 26.3%, and 61.3% across obstruction grades. Among children with complete obstruction, fibrotic mucosa was associated with higher OSA-18 scores than edematous mucosa (82.3 vs. 76.8; *p* = 0.02). *Conclusions*: ET obstruction grade independently determines audiological and quality-of-life outcomes in pediatric chronic adenoiditis. Mucosal remodeling further amplifies quality-of-life burden in complete obstruction. These findings support routine ET endoscopic grading in pediatric otorhinolaryngology risk stratification.

## 1. Introduction

Chronic adenoiditis is among the most prevalent inflammatory conditions of early childhood, frequently co-occurring with otitis media with effusion (OME) and Eustachian tube dysfunction (ETD) [1,2]. The adenoid tissue occupies the nasopharyngeal vault in close anatomical proximity to the Eustachian tube orifice, and its enlargement can compromise tubal ventilation through mechanical obstruction, mucosal inflammation, and biofilm-mediated chronic infection [3,4]. These processes collectively impair middle ear pressure equalization, predisposing to negative intratympanic pressure, effusion accumulation, and conductive hearing loss [5].

The clinical impact of chronic adenoiditis extends beyond audiological consequences. Adenoidal hypertrophy is a leading cause of upper airway obstruction in children, contributing to sleep-disordered breathing, impaired sleep quality, neurocognitive difficulties, and global health-related quality-of-life deterioration [6,7]. The OSA-18 questionnaire is a validated pediatric instrument specifically designed to quantify the impact of sleep-disordered breathing on health-related quality of life across multiple domains, including sleep disturbance, physical suffering, emotional distress, daytime problems, and caregiver concerns [8]. Its systematic use in clinical research allows for the objective comparison of functional burden across patient subgroups.

Traditional clinical assessment of adenoidal disease has relied predominantly on anatomical grading of nasopharyngeal obstruction, most commonly using the Cassano classification, which grades adenoid hypertrophy based on the percentage of choanal obstruction observed during flexible nasopharyngoscopy [9]. While this system provides a useful standardized framework, it does not specifically characterize the degree to which the Eustachian tube orifice is compromised. Endoscopic evaluation of ET orifice obstruction—classifiable as none, partial, or complete—provides a more direct measure of the functional threat to middle ear ventilation and has been proposed as a clinically meaningful parameter in pediatric otorhinolaryngology [10].

Although the association between adenoidal hypertrophy and middle ear dysfunction has been extensively documented [1,2,5], most clinical studies have relied on anatomical grading systems such as the Cassano classification as the primary measure of disease severity [9]. Whether the specific degree of ET orifice obstruction explains variance in audiological and quality-of-life outcomes beyond what is captured by overall choanal obstruction grade—and independently of systemic inflammatory markers, allergic status, and infection frequency—has not been formally addressed in the existing literature. This distinction has direct clinical relevance: if ET orifice obstruction grade contributes independent predictive information, its systematic documentation during nasopharyngoscopy could refine risk stratification and better inform the timing of surgical intervention beyond what adenoidal size grading alone can provide [9,10]. To our knowledge, this is the first study to quantify the independent contribution of endoscopically assessed ET orifice obstruction grade to audiological and quality-of-life outcomes after adjustment for anatomical, inflammatory, and allergic confounders, and the first to examine whether mucosal appearance modifies this relationship.

Additionally, the mucosal appearance of the adenoid tissue and ET region—specifically whether it is predominantly edematous or fibrotic/remodeling—may further modulate functional outcomes. Fibrotic remodeling represents a late-stage structural change reflecting chronic inflammatory activity and is likely to have a more pronounced and persistent effect on tubal function than reversible edematous changes [11]. Whether mucosal appearance modifies the relationship between ET obstruction and quality-of-life burden has not been formally evaluated.

The present study addresses these gaps by analyzing a well-characterized retrospective cohort of 236 pediatric patients with chronic adenoiditis and OME, in whom ET orifice obstruction was systematically graded endoscopically alongside clinical, audiological, and inflammatory parameters. We conducted a focused analysis with ET obstruction grade as the primary exposure, and PTA, MEP, OSA-18, and persistent middle ear dysfunction (PMED) as primary outcomes. The aims were (1) to characterize audiological and quality-of-life profiles across ET obstruction grades; (2) to evaluate the independent association of ET obstruction score with PTA, PTA > 30 dB, and OSA-18 total score in adjusted multivariate models; and (3) to assess the modifying role of mucosal appearance on quality-of-life outcomes among children with complete ET obstruction.

## 2. Materials and Methods

### 2.1. Study Design and Ethical Approval

This was a retrospective observational cohort analysis conducted at the Department of Otorhinolaryngology of the County Emergency Clinical Hospital Bihor and affiliated outpatient clinics, covering the period from 1 January 2022 to 31 December 2023. Clinical and paraclinical data were extracted retrospectively from institutional electronic medical records after completion of follow-up. All procedures and follow-up decisions were performed according to standard clinical practice guidelines. The study was conducted in accordance with the Declaration of Helsinki and was approved by the institutional Ethics Committee (Approval No. 39010/12.12.2024). Given the retrospective design and fully anonymized data processing, the requirement for individual informed consent was waived.

### 2.2. Study Population

Of 289 patients initially screened, 236 children aged 3–12 years were included after applying pre-specified inclusion and exclusion criteria. Inclusion required: a clinical diagnosis of chronic adenoiditis (nasopharyngeal symptoms persisting > 12 weeks); documented OME confirmed by otoscopy and type B or C tympanogram; complete laboratory, endoscopic, and audiological data at baseline; and a minimum follow-up of six months. Patients were excluded if they presented with craniofacial malformations, primary or secondary immunodeficiency, previous otologic surgery, tympanic membrane perforation, acute suppurative otitis media at baseline, or incomplete medical records. The final cohort comprised 128 males (54.2%) and 108 females (45.8%), with a mean age of 6.8 ± 2.1 years. Severe adenoidal hypertrophy (Cassano grade III–IV) was present in 127 patients (53.8%), and allergic status was documented in 100 children (42.4%).

### 2.3. Endoscopic Evaluation and Eustachian Tube Obstruction Grading

Flexible nasopharyngoscopy was performed under topical anesthesia using a pediatric fiberoptic endoscope by experienced otorhinolaryngologists following a standardized institutional protocol. Adenoidal hypertrophy was graded according to the Cassano classification (Grades I–IV), based on the percentage of choanal obstruction [9]. ET orifice obstruction was graded as: none (ET orifice fully visible and unobstructed), partial (ET orifice partially covered by adenoidal tissue, with residual lumen visible), or complete (ET orifice entirely obscured). For regression modeling, this ordinal variable was encoded as a numeric score (0 = none, 1 = partial, 2 = complete). To assess the reliability of this grading, a random subset of 40 recorded examinations was re-graded independently by a second otorhinolaryngologist and re-graded by the original examiner after an interval of at least four weeks; agreement was high for both inter-observer (weighted κ = 0.84) and intra-observer (weighted κ = 0.89) assessments. Additionally, mucosal appearance was recorded as either predominantly edematous or fibrotic/remodeling, and the presence of mucopurulent secretion was noted. Edematous mucosa was defined endoscopically as a glistening, pale-to-erythematous, swollen surface with preserved smooth contour, whereas fibrotic/remodeling mucosa was defined as a dull, pale, thickened, and irregular or scarred surface with loss of the normal glistening appearance and reduced pliability on instrument contact.

### 2.4. Audiological and Clinical Assessment

Pure tone audiometry was performed by certified audiologists using an Inventis Harp clinical diagnostic audiometer (Inventis S.r.l., Padova, Italy) in a sound-treated booth, calibrated to ISO 8253-1 [12] and ANSI S3.6 [13] standards. The pure tone average (PTA) was calculated as the mean air conduction threshold at 500, 1000, and 2000 Hz; in patients with unilateral OME the affected ear was used, whereas when bilateral OME was present, the worse-hearing ear was used. Tympanometry, classified as type A, B, or C per the Jerger system [14], was performed with an Inventis Timpani clinical impedance audiometer (Inventis S.r.l., Padova, Italy) using a 226 Hz probe tone, and was used for diagnostic confirmation and outcome definition. Middle ear pressure (MEP, in daPa) was recorded for each ear, and the worst value was used for analysis. Clinically relevant hearing loss was defined as PTA > 30 dB. This threshold was selected because a PTA exceeding 30 dB corresponds to a moderate degree of conductive hearing loss that is consistently associated with measurable impairment of speech perception and language development in children and approximates the level at which surgical intervention is commonly considered in pediatric OME [15,16].

Health-related quality of life was assessed using the OSA-18 questionnaire, a validated 18-item pediatric instrument yielding a total score of 18–126, with higher scores indicating greater impairment. Severe quality-of-life impairment was defined as an OSA-18 total score > 60 [8]. Clinical variables included age, sex, symptom duration (months), and the number of acute otitis media (AOM) episodes per year. Allergic status was defined by documented positive skin prick test and/or elevated total IgE according to age-adjusted reference values. Hematologic inflammatory markers (NLR, eosinophil count, CRP, total IgE) were collected from peripheral blood samples obtained within two weeks of the otorhinolaryngological assessment.

### 2.5. Outcome Definition

The primary outcomes were: (1) PTA as a continuous variable; (2) PTA > 30 dB as a binary outcome; (3) OSA-18 total score as a continuous variable; and (4) persistent middle ear dysfunction (PMED) at six months. PMED was defined as the presence of at least one of: persistent type B or C tympanogram; PTA > 25 dB; or indication for tympanostomy tube placement at six-month follow-up.

### 2.6. Statistical Analysis

Distribution normality for continuous variables was assessed using the Shapiro–Wilk test prior to parametric analysis. Continuous variables following a normal distribution were expressed as mean ± standard deviation (SD); non-normally distributed variables were reported as median and interquartile range (IQR). Categorical variables were reported as absolute frequencies and percentages. Between-group comparisons across ET obstruction grades (none, partial, complete) used one-way ANOVA for normally distributed continuous variables and the Kruskal–Wallis test for non-normally distributed variables; chi-squared test was applied for categorical variables. Post hoc pairwise comparisons were performed using independent samples *t*-tests with Bonferroni correction for multiple comparisons. Pearson correlation coefficients were computed to quantify bivariate associations between ET obstruction score and outcome measures.

Three multivariate models were constructed with ET obstruction score as the primary predictor, adjusting for age, sex, Cassano grade, neutrophil-to-lymphocyte ratio (NLR), allergic status, and acute otitis media (AOM) episodes per year: Covariates were selected a priori on clinical and pathophysiological grounds rather than by data-driven stepwise procedures: age and sex as standard demographic confounders; Cassano grade to account for overall anatomical adenoidal burden; NLR as a marker of systemic inflammatory activity; allergic status to capture atopic contribution to mucosal disease; and AOM episode frequency as an index of recurrent infectious load. We also assessed the following: (1) multiple linear regression with PTA as the continuous outcome, with model fit reported as adjusted R^2^; (2) binary logistic regression with PTA > 30 dB as the outcome, with discrimination evaluated using the area under the receiver operating characteristic curve (AUC), and calibration assessed using the Hosmer–Lemeshow goodness-of-fit test and Nagelkerke R^2^; and (3) multiple linear regression with OSA-18 total score as the continuous outcome, with model fit reported as adjusted R^2^. Variance inflation factors (VIF) were computed to assess multicollinearity among predictors; a VIF > 5 was considered indicative of significant collinearity. Within the subgroup of children with complete ET obstruction, independent samples *t*-tests were used to compare PTA and OSA-18 scores between those with fibrotic and edematous mucosal appearance. All statistical analyses were conducted using R (version 4.3.0) and Python with the statsmodels library (version 0.14). A two-tailed *p*-value < 0.05 was considered statistically significant.

## 3. Results

### 3.1. Cohort Characteristics and ET Obstruction Distribution

A total of 236 children were included (mean age 6.8 ± 2.1 years; 54.2% male). ET orifice obstruction was absent in 42 children (17.8%), partial in 114 (48.3%), and complete in 80 (33.9%). The overall mean PTA was 30.2 ± 7.8 dB, mean OSA-18 total score was 66.4 ± 16.0, and mean MEP was −137.9 ± 47.4 daPa. PMED at six months was observed in 82 children (34.7%). Severe adenoidal hypertrophy (Cassano grade III–IV) was documented in 127 patients (53.8%), and allergic status was confirmed in 100 patients (42.4%). Baseline characteristics stratified by ET obstruction grade are presented in Table 1. In clinical terms, audiological, middle-ear, and quality-of-life burden all increased in a stepwise manner across the three obstruction grades, with children showing complete ET obstruction concentrating the greatest proportion of severe hearing loss, markedly negative middle ear pressure, and persistent middle ear dysfunction at follow-up—a gradient that identifies complete obstruction as the subgroup at highest risk and most likely to warrant prioritized intervention.

### 3.2. Bivariate Correlations Between ET Obstruction Score and Outcome Measures

**Figure 1:**
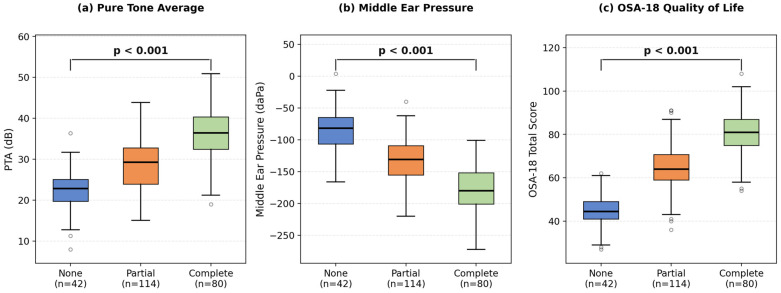
Distribution of (**a**) pure tone average (PTA), (**b**) middle ear pressure (MEP), and (**c**) OSA-18 total score across Eustachian tube obstruction grades (none, partial, complete). Boxes represent interquartile range; horizontal lines indicate medians; whiskers extend to 1.5× IQR. All between-group differences were significant at *p* < 0.001 (one-way ANOVA with post hoc pairwise comparisons). Higher PTA and OSA-18 values indicate worse hearing and greater quality-of-life impairment, respectively, while more negative MEP indicates poorer middle ear ventilation. Across all three measures, impairment increased progressively from the none to the complete obstruction group (n = 42, 114, and 80 children, respectively). PTA and MEP are expressed in dB and daPa; OSA-18 ranges from 18 to 126.

ET obstruction score showed strong positive correlations with PTA (r = 0.629, *p* < 0.001) and OSA-18 total score (r = 0.766, *p* < 0.001), and a strong negative correlation with MEP (r = −0.707, *p* < 0.001). PTA and OSA-18 were also significantly correlated with each other (r = 0.515, *p* < 0.001). Post hoc pairwise comparisons confirmed that all three obstruction grades differed significantly from each other on all primary outcome measures (all adjusted *p* < 0.001). The distribution of PTA, MEP, and OSA-18 scores across ET obstruction grades is illustrated in Figure 1.

**Figure 2:**
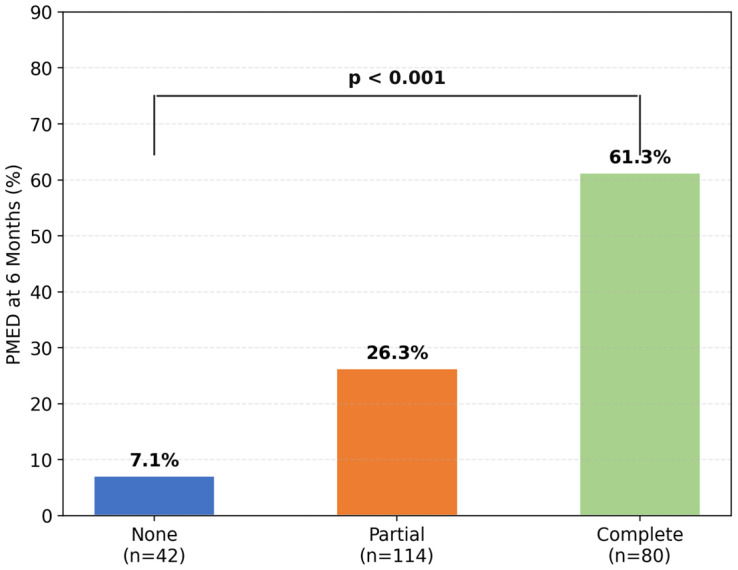
Prevalence of persistent middle ear dysfunction (PMED) at six months of follow-up by Eustachian tube obstruction grade. Percentages above bars indicate group-specific rates. Chi-squared test across groups: *p* < 0.001. PMED was defined as the presence at six months of at least one of: persistent type B or C tympanogram, PTA > 25 dB, or an indication for tympanostomy tube placement. Rates were 7.1% (none), 26.3% (partial), and 61.3% (complete), demonstrating a marked stepwise increase in persistent dysfunction with greater obstruction severity.

The prevalence of persistent middle ear dysfunction increased markedly with ET obstruction severity: 7.1% in the none group, 26.3% in the partial group, and 61.3% in the complete group (Figure 2; χ^2^ = 42.47, *p* < 0.001).

**Figure 3:**
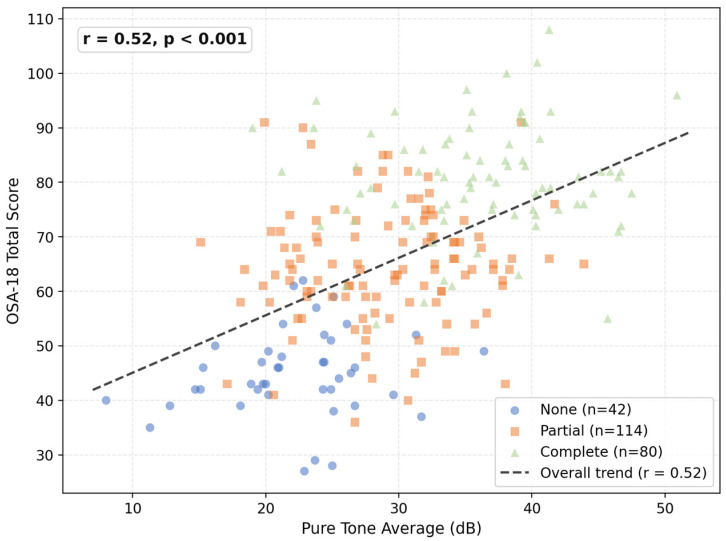
Scatter plot of pure tone average (PTA) versus OSA-18 total score, stratified by Eustachian tube obstruction grade. The dashed line represents the overall regression trend (r = 0.52, *p* < 0.001). Each point represents one patient; color and shape indicate ET obstruction grade. Points in the upper-right region correspond to children with both elevated PTA (worse hearing) and elevated OSA-18 (greater quality-of-life impairment); the complete-obstruction group clusters predominantly in this region, whereas the none group clusters in the lower-left, illustrating the joint progression of audiological and quality-of-life burden with increasing obstruction grade.

The correlation between PTA and OSA-18 score, displayed across ET obstruction grades, is shown in Figure 3. The scatter plot illustrates how both audiological and quality-of-life impairment intensify with increasing obstruction grade, with the complete obstruction group clustering in the upper-right quadrant (elevated PTA and elevated OSA-18).

### 3.3. Multivariate Regression Models

Results of the three multivariate models are presented in Table 2. In the linear regression model for PTA, ET obstruction score was the strongest independent predictor (β = 5.57, 95% CI 4.37–6.77; *p* < 0.001), indicating that each grade increment in ET obstruction was associated with a 5.57 dB increase in PTA after full covariate adjustment. NLR (β = 1.62; *p* = 0.001) and AOM episodes per year (β = 1.11; *p* < 0.001) also independently predicted higher PTA. Cassano grade showed a borderline association (β = 0.82; *p* = 0.073), while age, sex, and allergic status were not significant. The model explained a substantial proportion of variance in PTA (adjusted R^2^ = 0.52). Multicollinearity among predictors was acceptable, with all variance inflation factors below the pre-specified threshold (all VIF < 2.5; highest for Cassano grade and ET obstruction score), confirming the absence of clinically relevant collinearity between ET obstruction grade and Cassano grade.

In the logistic model for PTA > 30 dB, ET obstruction score remained the dominant predictor (OR = 5.12, 95% CI 2.84–9.24; *p* < 0.001), followed by NLR (OR = 2.23; *p* < 0.001) and AOM episodes (OR = 1.41; *p* = 0.008). The model achieved an AUC of 0.86, indicating excellent discriminative ability. Calibration was adequate, with a non-significant Hosmer–Lemeshow goodness-of-fit test (χ^2^ = 6.41, df = 8, *p* = 0.60), and the model accounted for a moderate-to-large share of outcome variance (Nagelkerke R^2^ = 0.46).

For OSA-18 total score, ET obstruction score exerted the largest effect (β = 14.89, 95% CI 12.87–16.92; *p* < 0.001), followed by allergic status (β = 4.24; *p* = 0.001), Cassano grade (β = 3.10; *p* < 0.001), and AOM episodes (β = 1.33; *p* = 0.005). NLR, age, and sex did not independently predict OSA-18 scores. This model demonstrated strong overall fit (adjusted R^2^ = 0.71), and all variance inflation factors again remained below the pre-specified threshold (all VIF < 2.5).

### 3.4. Effect of Mucosal Appearance Among Children with Complete ET Obstruction

Among the 80 children with complete ET obstruction, 52 (65.0%) demonstrated fibrotic/remodeling mucosal appearance and 28 (35.0%) showed predominantly edematous mucosa. Comparisons between the two subgroups are presented in Table 3.

**Figure 4:**
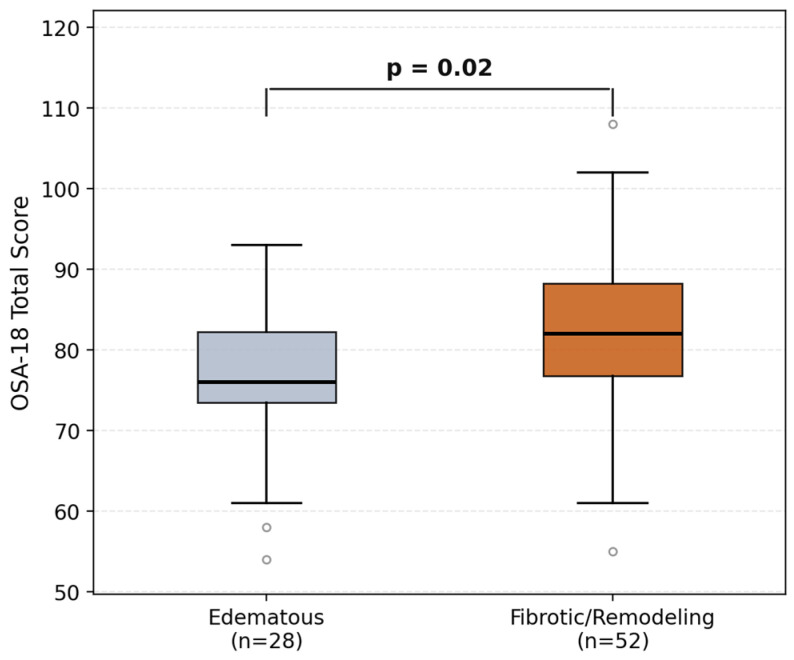
OSA-18 total score by mucosal appearance (edematous vs. fibrotic/remodeling) among children with complete Eustachian tube obstruction (n = 80). Boxes represent interquartile range; horizontal lines indicate medians. Independent samples *t*-test: *p* = 0.02. Subgroups comprised 28 children with edematous and 52 with fibrotic/remodeling mucosa. Despite comparable hearing thresholds between subgroups (*p* = 0.665), the fibrotic subgroup had significantly higher OSA-18 scores (82.3 ± 9.7 vs. 76.8 ± 11.1), indicating greater quality-of-life burden associated with mucosal remodeling within the same obstruction grade.

PTA did not differ significantly between children with fibrotic and edematous mucosa (36.4 ± 7.0 dB vs. 35.7 ± 6.2 dB; *p* = 0.665), indicating that mucosal appearance does not further modify hearing thresholds beyond the effect of complete ET obstruction itself. However, OSA-18 scores were significantly higher in the fibrotic subgroup compared to the edematous subgroup (82.3 ± 9.7 vs. 76.8 ± 11.1; *p* = 0.020), indicating that fibrotic remodeling is associated with greater quality-of-life burden even within the same obstruction grade. The difference in OSA-18 between mucosal subgroups is illustrated in Figure 4.

## 4. Discussion

The present study demonstrates that ET orifice obstruction grade is a strong, independent determinant of audiological outcomes and health-related quality of life in children with chronic adenoiditis and OME. After adjustment for Cassano grade, NLR, allergic status, age, sex, and AOM episode frequency, each increment in ET obstruction score was associated with a 5.57 dB increase in PTA and a 14.89-point increase in OSA-18 total score. These effect sizes are clinically meaningful: a difference of approximately 5 dB in PTA can alter speech perception in classroom settings [15,16], and an OSA-18 increment of approximately 15 points corresponds to a clinically significant change in quality-of-life burden [8,17]. The adjusted odds of clinically relevant hearing loss (PTA > 30 dB) were more than five-fold higher per obstruction increment, and PMED at six months occurred in 61.3% of children with complete obstruction versus 7.1% in those with no obstruction.

A particularly important finding is that ET obstruction grade retained independent predictive value after adjustment for Cassano grade, which itself did not significantly predict PTA in the multivariate model. This dissociation suggests that the degree of choanal obstruction does not fully capture the functional threat to Eustachian tube patency, and that direct endoscopic assessment of the ET orifice provides clinically non-redundant information. These findings are consistent with prior observations that anatomical grading of adenoid size correlates imperfectly with audiological outcomes and that ET dysfunction may occur even in the absence of marked choanal obstruction [5,18]. The present data suggest that endoscopic ET grading should be considered a distinct and complementary parameter in the clinical evaluation of children with chronic adenoiditis [10].

The independent contribution of NLR to PTA in the multivariate model is consistent with broader evidence suggesting that systemic neutrophil-dominant inflammation contributes to mucosal edema and impaired tubal ventilation [19,20,21]. Elevated NLR has been proposed as a marker of innate immune activation and chronic mucosal inflammation, both of which may sustain tubal dysfunction beyond the direct mechanical effect of adenoidal enlargement. This finding reinforces the concept that audiological outcomes in pediatric OME are determined not only by structural factors but also by the systemic inflammatory milieu. By contrast, allergic status independently predicted OSA-18 but not PTA, suggesting that atopic sensitization exerts a greater influence on upper airway functional burden and sleep quality than on middle ear ventilation per se. This differential pattern is biologically plausible: Th2-driven mucosal edema predominantly affects the nasopharyngeal and turbinate mucosa, contributing to nasal obstruction and sleep-disordered breathing, while direct tubal inflammation appears to require additional structural or infectious co-factors [22,23].

The modifying effect of mucosal appearance on OSA-18 scores within the complete ET obstruction subgroup represents a novel finding of this study. Children with fibrotic/remodeling mucosa had significantly higher OSA-18 scores than those with edematous mucosa (82.3 ± 9.7 vs. 76.8 ± 11.1; *p* = 0.02), despite comparable hearing thresholds. This dissociation suggests that fibrotic remodeling contributes to quality-of-life impairment through mechanisms beyond its effect on middle ear ventilation, potentially including increased upper airway stiffness [24], reduced mucociliary clearance [25], and greater chronic nasal obstruction, each of which may independently worsen sleep quality and daytime functioning [26,27]. The absence of a corresponding difference in PTA between mucosal subgroups further indicates that, once complete ET obstruction is established, the degree of structural remodeling influences functional well-being through pathways that are partially dissociated from the audiological pathway [28]. 

From a clinical perspective, the present findings support the routine documentation of ET orifice obstruction grade during nasopharyngoscopy in children with chronic adenoiditis [5,29]. The graded relationship between ET obstruction and both audiological and quality-of-life outcomes provides a rational basis for using this parameter to stratify intervention urgency [9]. Children with complete ET obstruction—particularly those with concurrent fibrotic mucosal appearance, elevated NLR, and frequent AOM episodes—may benefit from earlier surgical referral [9,19]. These findings also underscore the importance of integrating quality-of-life assessment through instruments such as the OSA-18 alongside audiological evaluation in the clinical management of pediatric adenoidal disease [2,30]. Concretely, these data suggest that the ET orifice grade could be recorded as a routine three-level descriptor (none/partial/complete) in every nasopharyngoscopy report, and that children with complete obstruction—in whom more than 80% had PTA > 30 dB and 61% had persistent middle ear dysfunction at six months—could be prioritized for audiometric and tympanometric reassessment and earlier consideration of adenoidectomy with or without tympanostomy tube placement, rather than relying on adenoid size alone. Conversely, children with absent or partial obstruction, low inflammatory markers, and edematous (rather than fibrotic) mucosa may be candidates for an initial period of watchful waiting with structured audiological surveillance.

Several limitations of this study must be acknowledged. The retrospective design limits causal inference and may introduce selection and information bias. Because only children with complete baseline laboratory, endoscopic, and audiological data and a minimum six-month follow-up were eligible, those with incomplete records were necessarily excluded; this may have favored more closely monitored or more severely affected patients and could limit how representative the cohort is of the wider population with chronic adenoiditis. Information on the magnitude and pattern of missing data prior to exclusion was not systematically retrievable, and no imputation was performed, so residual selection bias cannot be excluded. In addition, although diagnostic and follow-up procedures followed standard institutional practice, therapeutic management during the six-month follow-up was not standardized across patients: differences in medical therapy and in the timing or use of surgical intervention may have influenced PMED, PTA, and OSA-18 outcomes, and the retrospective dataset did not permit a controlled analysis of treatment effects. Endoscopic ET grading, while performed by experienced otorhinolaryngologists using a standardized protocol, carries inherent inter-observer variability, although the high inter- and intra-observer agreement observed in the reliability subset (weighted κ = 0.84 and 0.89, respectively) supports the reproducibility of the grading system in this setting. The single-center setting limits generalizability, and external validation of the regression models in independent cohorts is required. As the cohort was drawn from a single tertiary referral department, the case mix may be skewed toward more severe or complex presentations, and the absolute event rates and effect estimates reported here may not transfer directly to primary-care or community settings or to populations with different demographic, environmental, or healthcare-access profiles; the directional gradient between ET obstruction grade and outcomes is, however, likely to be more broadly generalizable than the precise point estimates. The dataset does not include molecular biomarkers or tissue-level characterization of mucosal remodeling, so the biological mechanisms underlying the observed associations remain inferred rather than experimentally confirmed. Longitudinal inflammatory biomarker monitoring was not available due to the retrospective design, and therapeutic management was individualized according to clinical indications, precluding a standardized evaluation of treatment response stratified by ET obstruction grade. Future prospective multicenter studies incorporating repeated endoscopic assessments, standardized audiological follow-up, and tissue-level biomarker analysis are needed to confirm these findings and to evaluate whether ET obstruction grade can serve as a reliable guide for therapeutic decision-making in clinical practice.

## 5. Conclusions

Eustachian tube orifice obstruction grade is an independent and clinically meaningful determinant of audiological and quality-of-life outcomes in children with chronic adenoiditis and OME, contributing predictive value beyond that provided by adenoidal size alone. Complete ET obstruction is associated with clinically significant hearing loss, markedly negative middle ear pressure, and severe quality-of-life impairment in the vast majority of affected children. Among children with complete obstruction, fibrotic mucosal remodeling further amplifies quality-of-life burden without additionally worsening hearing thresholds, pointing to distinct mechanisms of functional deterioration in this sub-group. NLR independently predicts audiological outcomes, while allergic status specifically influences quality of life, supporting a multidimensional approach to risk characterization. These findings support the integration of routine ET orifice grading and OSA-18 assessment into the standard evaluation protocol for pediatric chronic adenoiditis, with the aim of improving individualized risk stratification and guiding timely therapeutic decision-making. Prospective multicenter validation is required before clinical implementation.

## Figures and Tables

**Table 1:** Baseline characteristics stratified by Eustachian tube obstruction grade (N = 236).

Variable	None (*n* = 42)	Partial (*n* = 114)	Complete (*n* = 80)	*p*-Value
Age (years), mean ± SD	6.9 ± 2.1	6.8 ± 2.1	6.7 ± 1.9	0.81
Male sex, *n* (%)	22 (52.4%)	56 (49.1%)	47 (58.8%)	0.50
Symptom duration (months), mean ± SD	11.9 ± 4.3	13.4 ± 4.0	16.9 ± 3.9	<0.001
AOM episodes/year, mean ± SD	1.64 ± 1.41	2.17 ± 1.40	2.35 ± 1.34	0.027
Cassano grade III–IV, *n* (%)	5 (11.9%)	60 (52.6%)	62 (77.5%)	<0.001
Allergic status, *n* (%)	12 (28.6%)	41 (36.0%)	41 (51.2%)	0.048
NLR, mean ± SD	2.53 ± 0.80	2.92 ± 0.77	3.24 ± 0.85	0.007
CRP (mg/L), mean ± SD	2.71 ± 0.94	3.00 ± 1.06	3.57 ± 1.07	<0.001
Total IgE (IU/mL), mean ± SD	100.4 ± 49.1	117.4 ± 54.2	127.2 ± 56.4	0.225
PTA (dB), mean ± SD	22.2 ± 5.5	29.0 ± 5.9	36.2 ± 6.7	<0.001
PTA > 30 dB, *n* (%)	3 (7.1%)	53 (46.5%)	65 (81.2%)	<0.001
MEP (daPa), mean ± SD	−82.1 ± 36.1	−130.5 ± 32.4	−177.7 ± 34.1	<0.001
OSA-18 total score, mean ± SD	44.9 ± 7.9	64.5 ± 11.0	80.4 ± 10.3	<0.001
OSA-18 > 60 (severe), *n* (%)	2 (4.8%)	77 (67.5%)	77 (96.2%)	<0.001
Mucopurulent secretion, *n* (%)	13 (31.0%)	51 (44.7%)	49 (61.3%)	0.004
Fibrotic mucosal appearance, *n* (%)	0 (0.0%)	9 (7.9%)	52 (65.0%)	<0.001
PMED at 6 months, *n* (%)	3 (7.1%)	30 (26.3%)	49 (61.3%)	<0.001

AOM: acute otitis media; CRP: C-reactive protein; IgE: immunoglobulin E; MEP: middle ear pressure; NLR: neutrophil-to-lymphocyte ratio; OSA-18: obstructive sleep apnea quality-of-life questionnaire; PMED: persistent middle ear dysfunction; PTA: pure tone average; SD: standard deviation. *p*-values from one-way ANOVA (continuous) or chi-squared test (categorical).

**Table 2:** Multivariate regression models for audiological and quality-of-life outcomes (N = 236).

Predictor	Estimate/OR	95% CI	*p*-Value	Model
Linear regression outcome: PTA (dB)				
ET obstruction score	β = 5.57	4.37–6.77	<0.001	LR-PTA
NLR	β = 1.62	0.68–2.57	0.001	LR-PTA
AOM episodes/year	β = 1.11	0.55–1.66	<0.001	LR-PTA
Cassano grade	β = 0.82	−0.08–1.72	0.073	LR-PTA
Allergic status	β = 0.23	−1.26–1.72	0.760	LR-PTA
Age	β = −0.11	−0.46–0.24	0.531	LR-PTA
Sex	β = 0.62	−0.87–2.10	0.415	LR-PTA
Logistic regression outcome: PTA > 30 dB (AUC = 0.86)				
ET obstruction score	OR = 5.12	2.84–9.24	<0.001	Log-PTA > 30
NLR	OR = 2.23	1.43–3.48	<0.001	Log-PTA > 30
AOM episodes/year	OR = 1.41	1.09–1.81	0.008	Log-PTA > 30
Cassano grade	OR = 1.37	0.93–2.03	0.111	Log-PTA > 30
Allergic status	OR = 0.76	0.39–1.49	0.424	Log-PTA > 30
Age	OR = 1.00	0.86–1.18	0.968	Log-PTA > 30
Sex	OR = 0.83	0.43–1.58	0.564	Log-PTA > 30
Linear regression outcome: OSA-18 total score				
ET obstruction score	β = 14.89	12.87–16.92	<0.001	LR-OSA18
Cassano grade	β = 3.10	1.59–4.62	<0.001	LR-OSA18
Allergic status	β = 4.24	1.73–6.76	0.001	LR-OSA18
AOM episodes/year	β = 1.33	0.40–2.26	0.005	LR-OSA18
NLR	β = 0.10	−1.50–1.70	0.898	LR-OSA18
Age	β = −0.43	−1.02–0.16	0.154	LR-OSA18
Sex	β = −0.67	−3.17–1.83	0.598	LR-OSA18

AOM: acute otitis media; AUC: area under the receiver operating characteristic curve; ET: Eustachian tube; Log-PTA > 30: binary logistic regression with PTA > 30 dB as outcome; LR-OSA18: multiple linear regression with OSA-18 total score as outcome; LR-PTA: multiple linear regression with PTA as outcome; NLR: neutrophil-to-lymphocyte ratio; OR: odds ratio; β: unstandardized regression coefficient. All models adjusted for age, sex, Cassano grade, NLR, allergic status, and AOM episodes per year.

**Table 3:** Audiological and quality-of-life outcomes by mucosal appearance in children with complete ET obstruction (n = 80).

Variable	Edematous (*n* = 28)	Fibrotic/Remodeling (*n* = 52)	*p*-Value
PTA (dB), mean ± SD	35.7 ± 6.2	36.4 ± 7.0	0.665
OSA-18 total score, mean ± SD	76.8 ± 11.1	82.3 ± 9.7	0.020
OSA-18 > 60 (severe), n (%)	27 (96.4%)	50 (96.2%)	0.960
PMED at 6 months, n (%)	18 (64.3%)	31 (59.6%)	0.691

ET: Eustachian tube; OSA-18: obstructive sleep apnea quality-of-life questionnaire; PMED: persistent middle ear dysfunction; PTA: pure tone average; SD: standard deviation. Comparisons by independent samples *t*-test (continuous) or chi-squared test (categorical).

## Data Availability

The raw data supporting the conclusions of this article will be made available by the authors on request.

## Abbreviations

The following abbreviations are used in this manuscript:

AOM	Acute Otitis Media
AUC	Area Under the Curve
CI	Confidence Interval
CRP	C-Reactive Protein
ET	Eustachian Tube
ETD	Eustachian Tube Dysfunction
IgE	Immunoglobulin E
MEP	Middle Ear Pressure
NLR	Neutrophil-to-Lymphocyte Ratio
OME	Otitis Media with Effusion
OR	Odds Ratio
OSA-18	Obstructive Sleep Apnea-18 Quality-of-Life Questionnaire
PMED	Persistent Middle Ear Dysfunction
PTA	Pure Tone Average
SD	Standard Deviation
